# Termite Detection Techniques in Embankment Maintenance: Methods and Trends

**DOI:** 10.3390/s25144404

**Published:** 2025-07-15

**Authors:** Xiaoke Li, Xiaofei Zhang, Shengwen Dong, Ansheng Li, Liqing Wang, Wuyi Ming

**Affiliations:** 1 Henan Key Lab of Intelligent Manufacturing of Mechanical Equipment, Zhengzhou University of Light Industry, Zhengzhou 450002, China; lixiaoke@zzuli.edu.cn (X.L.); 332404040371@zzuli.edu.cn (X.Z.); 2 Key Laboratory of Termite Control of Ministry of Water Resources, Hubei Water Resources Research Institute, Wuhan 430070, China; 3 Hydropower Equipment and Intelligent System Engineering Technology Research Centre of Henan Province, Department of Mechanical and Electronic Engineering, Henan Vocational College of Water Conservancy and Environment, Zhengzhou 450002, China; liansheng@zzuli.edu.cn (A.L.); wangliqing0225@163.com (L.W.); 4 Guangdong Provincial Key Laboratory of Digital Manufacturing Equipment, Huazhong University of Science and Technology, Dongguan 523808, China; mingwuyi@zzuli.edu.cn

**Keywords:** embankment, termites, detection, physical sensing, biological characteristics

## Abstract

Termites pose significant threats to the structural integrity of embankments due to their nesting and tunneling behavior, which leads to internal voids, water leakage, or even dam failure. This review systematically classifies and evaluates current termite detection techniques in the context of embankment maintenance, focusing on physical sensing technologies and biological characteristic-based methods. Physical sensing methods enable non-invasive localization of subsurface anomalies, including ground-penetrating radar, acoustic detection, and electrical resistivity imaging. Biological characteristic-based methods, such as electronic noses, sniffer dogs, visual inspection, intelligent monitoring, and UAV-based image analysis, are capable of detecting volatile compounds and surface activity signs associated with termites. The review summarizes key principles, application scenarios, advantages, and limitations of each technique. It also highlights integrated multi-sensor frameworks and artificial intelligence algorithms as emerging solutions to enhance detection accuracy, adaptability, and automation. The findings suggest that future termite detection in embankments will rely on interdisciplinary integration and intelligent monitoring systems to support early warning, rapid response, and long-term structural resilience. This work provides a scientific foundation and practical reference for advancing termite management and embankment safety strategies.

## 1. Introduction

As a critical component of water conservancy infrastructure, embankments play an essential role in flood control and disaster mitigation, agricultural irrigation, urban and rural water supply, water resource regulation, and ecosystem maintenance. The operational safety of embankments is directly linked to regional socio-economic development and the protection of human life and property. However, due to environmental changes, construction quality variations, material aging, and biological erosion, embankment structures are exposed to complex and diverse safety risks. Among these, termites pose a significant hidden threat by constructing concealed nests and tunnels within embankments, leading to localized voids, soil loosening, and structural weakening [1,2,3]. Consequently, incorporating termite activity monitoring into embankment safety management and conducting scientific, efficient detection and mitigation efforts have become critical technical challenges and key research directions for ensuring the long-term stability and functionality of embankment projects.

**Figure 1:**
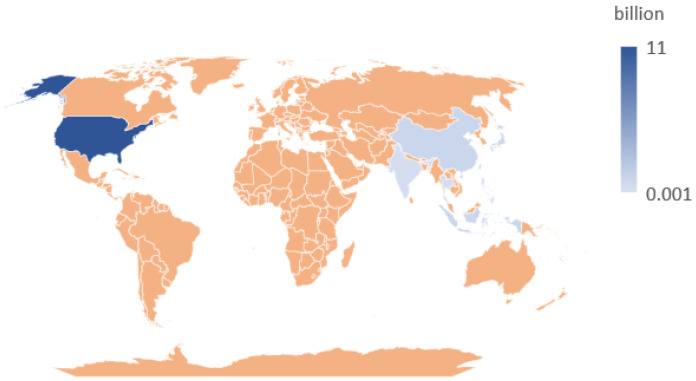
Countries with the most severe termite-related losses [4,10,11,12,13,14,15,16,17].

Termites are small, socially organized insects that live in colonies, and their activities pose a serious threat to infrastructure and property worldwide [4,5,6,7]. Studies show that subterranean termites account for approximately 80% of termite-related damage, with global economic losses as high as USD 50 billion [8,9]. Countries with the most severe termite-related losses are identified in Figure 1. In southern China, approximately 90% of embankments and reservoirs suffer annual damage from termites, resulting in economic losses exceeding USD 1 billion, with Indonesia experiencing comparable levels of damage. In India, termite-related damages amount to approximately USD 35.12 million each year. Japan spends between USD 800 million and 1 billion annually on termite prevention and control. In the United States, economic losses reach up to USD 11 billion, with termite-induced property damage in some areas surpassing the combined impact of storms and fires.

The downstream slopes of embankments are typically covered with vegetation, while the upstream slopes often accumulate floating debris. Therefore, termite colonies are attracted from nearby hills, where they search for food and nesting sites. The suitable soil, abundant water sources, and favorable temperature and humidity conditions in the embankment area provide an ideal habitat for termites [18]. Termites rapidly invade the interior of embankments through underground tunnels and surface cracks, constructing complex nests and shelters while feeding on cellulose found in plants [19,20,21]. These nests consist of winding tunnels and internal cavities, which severely compromise the structural stability of the embankment and affect its safety [22,23]. The cavities and multi-channel structures formed by termites weaken the embankment’s water-retaining capability, leading to issues such as leakage and settlement [24,25,26]. In cases of severe infestation, termite damage may trigger catastrophic events such as embankment failure or collapse [4,27,28].

The potential threat of termites to embankment structures and their global impact urgently requires the adoption of advanced detection methods for timely and effective monitoring and intervention. In addition to traditional visual inspection, two primary categories of non-destructive termite detection technologies have emerged in recent years including physical detection and biological sensing technologies. Within the domain of physical methods, acoustic emission techniques are capable of capturing transient signals generated by termite activity such as chewing or movement [29,30]. When combined with spectral kurtosis analysis and wavelet-based denoising, these methods significantly enhance signal extraction under low signal-to-noise conditions [31]. Ground-penetrating radar (GPR) analyzes the reflection of electromagnetic waves within the soil to detect termite-induced cavities, moisture anomalies, and subsurface disturbances [2,32]. Additionally, by measuring spatial variations in subsurface resistivity electrical resistivity tomography (ERT) can reveal termite foraging tunnels, moisture-rich zones, and localized structural degradation [33,34]. This technique is particularly suitable for high-moisture levees and offers strong applicability for mapping large-scale termite-affected regions. In terms of biological sensing, chemical detection methods, such as gas chromatography and electronic nose systems, target volatile organic compounds (VOCs) emitted by termites, including compounds like naphthalene and 2-phenoxyethanol [35,36]. Sensor arrays composed of conductive polymers (CPs), metal oxide semiconductors, and quartz crystal microbalances (QCMs) have been widely used to construct olfactory fingerprints for termite species classification and activity assessment [37].

Although these methods have shown promise in experimental and controlled settings, they often suffer from limitations such as restricted spatial coverage, sensitivity to environmental factors, and the need for close-proximity deployment [38]. These constraints limit their effectiveness in large-scale applications such as embankment monitoring. Consequently, the development of intelligent, scalable, and automated multi-sensor monitoring frameworks, supported by UAV platforms and AI-driven analytics, represents a critical direction for advancing termite detection in complex infrastructure environments.

**Figure 2:**
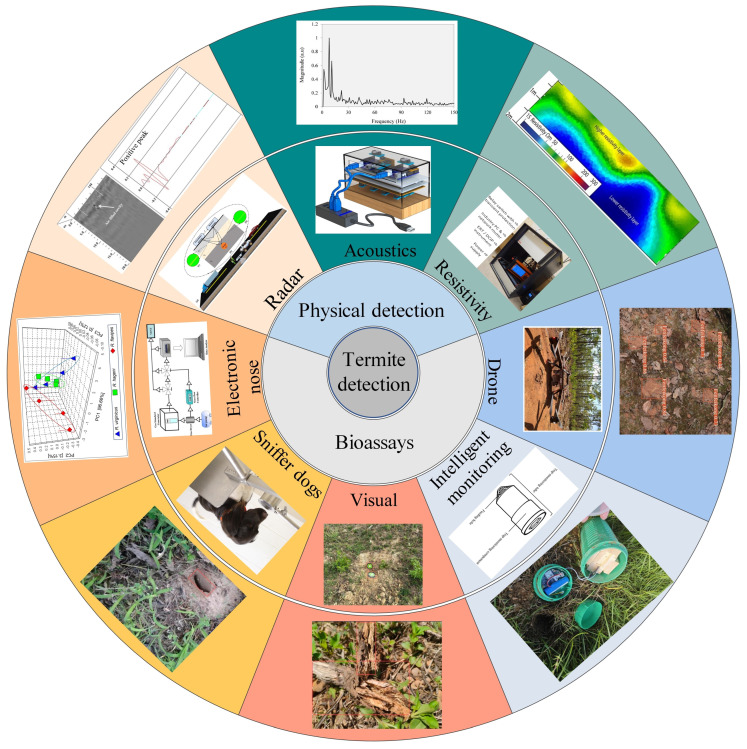
Different methods for termite detection [31,35,40,41,42,43,44,45,46,47].

As shown in Figure 2, termite detection technologies can be categorized into physical sensing technologies and biological characteristic detection methods. Physical sensing technologies include GPR, acoustic detection, and resistivity methods, which identify termite activity by analyzing changes in the internal structure of the embankment. Biological characteristic detection methods, on the other hand, involve gas detection and activity sign detection. Gas detection technologies, such as electronic noses and sniffer dogs, identify characteristic gases from termite metabolic products. Activity sign detection technologies employ visual inspection, intelligent monitoring, and drone image analysis to detect termite activity on the embankment surface. Based on the Technology Readiness Level framework proposed by the National Aeronautics and Space Administration, technologies are categorized into nine levels, ranging from initial conceptualization to full deployment, to systematically assess their maturity [39]. Several termite detection methods are considered highly mature (TRL 9), including GPR, electrical resistivity, acoustic sensing, sniffer dogs, and visual inspection—all of which have been successfully applied in real-world embankment or structural monitoring scenarios. In contrast, electronic noses and UAV-based image analysis are at a moderate maturity level (TRL 5), indicating successful laboratory validation but limited large-scale field application. Intelligent monitoring systems that integrate multi-sensor data and AI algorithms are currently assessed at TRL 7, having demonstrated functionality in operational environments but still requiring further standardization and optimization.

Recent advances in artificial intelligence provide new pathways for improving the automation and accuracy of termite detection. Deep learning models, such as convolutional neural networks, have been applied to analyze complex GPR signals for identifying subsurface anomalies, while object detection algorithms like YOLO and Mask R-CNN have enabled automatic recognition of termite mounds or thermal anomalies in UAV imagery [48]. Furthermore, AI-driven data fusion frameworks allow for real-time integration of multi-sensor inputs—such as acoustic signals, VOCs, and remote sensing data—enhancing the reliability of detection outcomes. These developments highlight the potential of incorporating standard machine learning tools into termite monitoring systems to enable scalable, intelligent embankment surveillance [42,49].

## 2. Physical Sensing Technologies

In termite detection of embankments, the use of advanced physical sensing technologies is crucial. These technologies, including ground-penetrating radar, acoustic detection, and resistivity methods, can effectively identify and locate termite activity and the structural damage it causes. Ground-penetrating radar works by emitting electromagnetic waves and analyzing the reflected signals, revealing anomalies within the embankment structure [50]. Acoustic detection utilizes the propagation characteristics of sound waves to monitor changes and abnormal vibrations within materials. The resistivity method detects structural changes caused by termite activity by measuring variations in the material’s electrical resistivity. These technologies provide precise and reliable detection tools for embankment maintenance, effectively ensuring the safety and stability of the embankment.

### 2.1. Ground-Penetrating Radar

GPR is a non-invasive method capable of real-time data collection and analysis. It plays a significant role in detecting and preventing underground voids and termite nests in embankments without causing any damage to the structure [1]. The effectiveness of GPR detection depends on several factors, including the mineral characteristics of the soil and sediments, clay content, soil moisture, target burial depth, topography, and vegetation conditions [51]. After years of technological development and practical application, GPR has become a highly mature detection tool, widely used in geological exploration [52], archaeological research [53], civil engineering [54], groundwater detection [55], and embankment internal structure monitoring [50]. As shown in Table 1, GPR has been applied in detecting cracks, animal burrows, and other structural anomalies.

**Figure 3:**
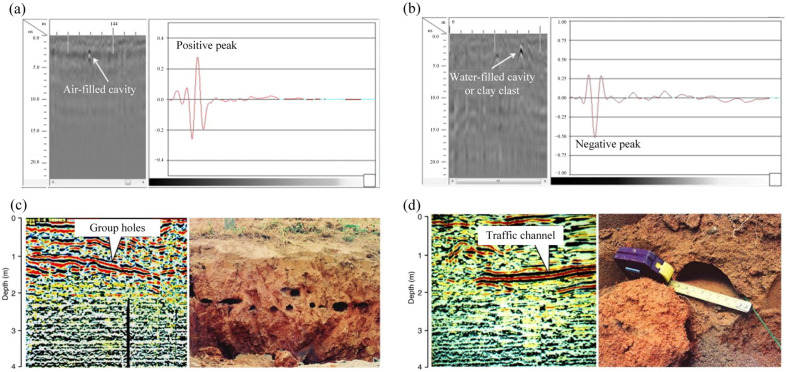
The application of GPR in the detection of termites. (**a**) The air-filled cavity profile detected by GPR of 900 MHz antenna [40]. (**b**) The water-filled cavity or clay-filled cavity profile detected by GPR of 900 MHz antenna [40]. (**c**) GPR images of termite group holes and verification obtained with a 500 MHz antenna [2]. (**d**) GPR images for termite traffic channel and verification obtained with a 500 MHz antenna [2].

GPR emits high-frequency electromagnetic waves and capture reflected signals from subsurface interfaces, which are subsequently processed to construct radar images [40]. Termite-induced anomalies, such as tunnels or voids, exhibit distinct dielectric and resistivity characteristics, significantly altering wave propagation velocities [58]. As illustrated in Figure 3a,b, these variations enable identification and depth estimation of cavities through waveform analysis and phase-axis tracking. Combined with the propagation velocity of electromagnetic waves in the ground, the depth of each reflective layer can be calculated using the following formula.

For media such as soil, rock, and freshwater, the propagation velocity V of electromagnetic waves can be expressed by Equation (1) [40]:

(1) $$V=C/\sqrt{\varepsilon_{\gamma }},$$

where C represents the speed of light under specific conditions, which is related to the dielectric constant of the medium, $\varepsilon_{\gamma }$ is the dielectric constant.

The distance D between the antenna and the reflection point can be expressed by Equation (2):

(2) $$D=V\cdot \frac{\Delta t}{2}$$

where Δt is the time taken for electromagnetic wave propagation from transmitter to receiver.

When an electromagnetic wave reaches the boundary between two dielectric materials, the degree of reflection depends on the difference in their dielectric constants. The reflection coefficient *R* at the interface between the two materials can be expressed by Equation (3) [2]:

(3) $$R=\frac{1-\sqrt{\varepsilon_{2}/\varepsilon_{1}}}{1+\sqrt{\varepsilon_{2}/\varepsilon_{1}}}$$

where $\varepsilon_{1}$ and $\varepsilon_{2}$ represent the dielectric constants of the two materials.

GPR has emerged as a promising non-invasive technique for detecting subsurface termite nests due to its ability to capture electromagnetic reflections from underground anomalies. The selection of an appropriate frequency range (10 MHz–1 GHz) is essential for detection performance. Specifically, higher frequencies provide enhanced resolution but suffer from shallow penetration depth, while lower frequencies offer greater penetration at the expense of image resolution [48]. As illustrated in Figure 3c,d, Xu et al. [2] conducted detection and verification of termite infestations in embankments, demonstrating that GPR technology can effectively identify both active termite nests and abandoned cavities left by deceased termites. The results confirmed that the method provides satisfactory real-time detection performance for both types of subsurface anomalies. Jiao et al. [48] proposed an automated termite nest detection framework by integrating GPR imaging with the YOLOv8 deep learning algorithm. In their study, both real-world GPR data collected from embankments and simulated GPR images containing termite nests, rocks, and voids are utilized for model training and validation. The YOLOv8 model demonstrated robust performance, achieving a mean average accuracy of 0.96 and a detection speed of 53.76 frames per second. These results underscore the potential of combining GPR with advanced deep learning-based object detection methods to significantly improve the accuracy, efficiency, and automation of termite nest identification in complex subsurface environments.

### 2.2. Acoustic Detection

**Figure 4:**
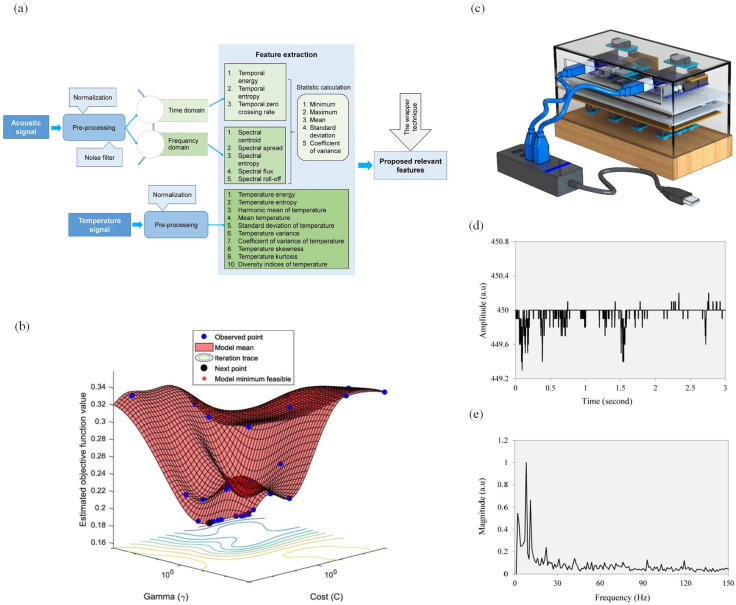
Acoustic-based detection techniques. (**a**) Detection flowchart [31]. (**b**) Optimal pairing selection [63]. (**c**) Detection equipment based on acoustic and temperature signals [31]. (**d**) Time-domain features [59]. (**e**) Frequency-domain features [59].

Acoustic detection is widely used in the field of insect detection [29], which utilizes acoustic sensors to capture sound, convert it into electrical signals, and amplify them to generate acoustic spectrograms. The process is shown in Figure 4a. The method is non-destructive, fast, and accurate. However, the precision of termite acoustic detection depends on several factors, such as the type of sensor and its frequency range, the properties of the substrate material, the contact interface between the sensor and the substrate, the body size and behavioral habits of the termites, as well as the spatial distance between the termites and the sensor [15]. Even simple termite activities produce sound signals. For instance, during feeding, movement, or tunneling, termites emit subtle sounds. When their nests or extended tunnels are disturbed, termites often generate alarm signals by tapping their heads, which is commonly referred to as “head-banging” [59,60,61,62]. Acoustic sensors can capture these sounds and convert them into amplified electrical or digital signals, which are ultimately processed into waveform diagrams by a signal processor.

Many studies have demonstrated that the most effective approach to distinguish termite acoustic emission signals from environmental noise involves the combined use of spectral kurtosis and wavelet transform techniques. Spectral kurtosis quantifies the peakedness of a signal’s frequency spectrum and is particularly effective in identifying non-Gaussian, impulsive components associated with termite activity, whereas environmental noise typically exhibits lower kurtosis and a more Gaussian distribution [45,64]. Building on this, the application of discrete wavelet transform or continuous wavelet transform enables multiscale decomposition of the signal. By evaluating wavelet entropy across decomposition levels, the scale corresponding to the lowest entropy and highest spectral kurtosis is selected as the optimal representation of the target signal [13]. Furthermore, the normalized acoustic signal can also be purified using a noise filter based on the threshold method to reduce the background noise [31].

Since acoustic signals can change rapidly over time, they are typically divided into individual frames for analysis. This segmentation has proven effective in detecting other insects, which is crucial for determining whether a signal originates from termites [31,65]. When termite activity is present nearby, acoustic peak patterns appear on the display. During such activity, a sudden spike in amplitude will immediately trigger an alarm. In contrast, the amplitude generated by feeding and tunneling behaviors is relatively low. By analyzing characteristic patterns of sound signals, termite activity levels can be visually classified [13]. De et al. [66] used SP-1L sensors to collect data and analyzed sliding cumulants and spectral kurtosis. The results showed that termite activity could be effectively detected in both the time and frequency domains.

In termite acoustic signal-based detection, the algorithms directly affect the detection accuracy [67]. De et al. [30] demonstrated that acoustic signals play a critical role in insect detection by using the ERICA algorithm to detect sounds produced by termites. In 2018, Nanda and colleagues extracted pure acoustic signals of termites but achieved an accuracy of only 83.75% due to environmental noise interference. The time-domain and frequency-domain features are shown in Figure 4d,e [59]. In 2019, they developed a novel termite detection system that integrated both acoustic and temperature signals, achieving an accuracy of 93.83%. The optimal parameter tuning process is illustrated in Figure 4b. By 2021, acoustic and temperature signal extraction was combined with the Boruta package to create a new detection model, as shown in Figure 4c, reaching an impressive detection accuracy of 97.167% and showing the ability to predict termite population size with a root mean squared error of 98.316 [31]. These findings highlight the substantial potential of acoustic and temperature signal extraction for detecting subterranean termites and predicting hidden infestations. With continuous improvements in detection equipment, modern signal processing technologies are becoming increasingly capable of extracting relevant signals from background noise, leading to higher accuracy rates [68]. However, acoustic devices are typically used for on-site detection and are highly sensitive to environmental conditions. Therefore, they cannot operate effectively in high-vibration environments, making them unsuitable for permanent monitoring [20].

### 2.3. Electrical Resistivity Method

The electrical resistivity method has emerged as a promising non-invasive technique for termite nest detection in embankments, owing to its capacity to distinguish subsurface materials based on variations in electrical conductivity. By placing multiple electrodes along a defined profile and injecting direct current into the ground, potential differences are measured and used to infer the spatial distribution of resistivity [69]. Termite nests, typically located in deeper layers and often associated with high-resistivity zones due to localized moisture retention and structural voids, produce distinct resistivity anomalies that can be effectively identified using this approach [70].

The typical workflow of resistivity-based monitoring methods includes data acquisition, signal filtering, and inversion calculations to produce high-resolution subsurface resistivity models. These models facilitate the identification of abnormal zones associated with termite activity and allow for the estimation of cavity size and distribution [71]. ERT, in particular, has been widely applied in engineering geology and dam safety evaluations [72,73,74,75].

Several field studies have demonstrated the efficacy of ERT in the context of termite detection. Loperte et al. [69] employed the Syscal R2 Switch48 resistivity system to investigate internal damage in the Sinni embankment in Italy, confirming the method’s effectiveness in delineating structural fractures. Weller et al. [33] utilized multi-electrode resistivity imaging at multiple depths and successfully localized termite nests. Excavation confirmed the presence of a primary chamber and three auxiliary cavities, validating the resistivity-based interpretation. Similarly, Dong et al. [76] applied two-dimensional resistivity imaging to the Wuhan embankments in China, successfully locating termite nests that were subsequently confirmed through excavation. These results demonstrate the method’s effectiveness for precise subsurface detection and spatial mapping of termite activity. However, the study by Hennig et al. [44] demonstrated that terrain conditions can affect measurement data in embankment termite detection, representing an important factor that must be considered during the monitoring process.

Compared to traditional visual inspection or invasive sampling, high-density resistivity imaging offers enhanced depth penetration, volumetric estimation, and coverage efficiency. It significantly improves the precision and reliability of termite detection in large-scale embankment systems. However, challenges such as the influence of heterogeneous soil conditions, noise sensitivity, and the need for complex inversion algorithms remain. Future research may benefit from integrating resistivity imaging with other geophysical or AI-based techniques to further improve the detection performance and operational efficiency.

### 2.4. Summary

**Figure 5:**
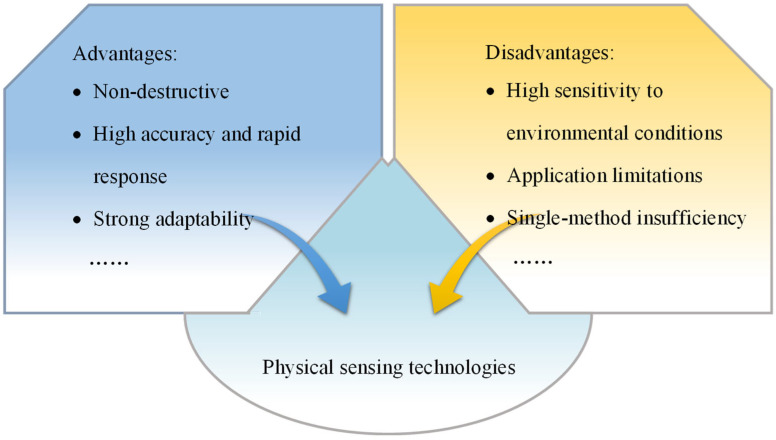
Advantages and disadvantages of physical sensing technologies.

Physical detection technologies offer notable advantages such as non-destructiveness, high accuracy, and rapid response. These methods primarily identify and locate subsurface anomalies caused by termite activity, which include techniques such as GPR, acoustic detection, and resistivity measurements. As shown in Table 2, we systematically evaluated several physical detection methods in key dimensions such as resolution, environmental limitations, cost, deployment complexity, and data interpretation. GPR detects spatial variations through the reflection of electromagnetic waves, enabling the localization of termite nests and tunnels without disturbing the embankment structure. It is well-suited for large-scale preliminary surveys but is sensitive to factors such as soil moisture content and conductivity. Acoustic detection analyzes the propagation characteristics of sound waves in soil, offering fast response and ease of implementation. However, it is vulnerable to external noise interference and is best applied in relatively quiet environments for localized inspections. Resistivity methods detect differences in soil electrical resistance to infer potential termite activity zones. This technique is most effective in geologically stable areas, though its detection depth and resolution are somewhat limited. In summary, physical detection methods demonstrate considerable adaptability and engineering value in practical applications, significantly enhancing both the coverage and depth of termite monitoring. A comparative summary of the advantages and limitations of typical physical detection methods is illustrated in Figure 5. However, due to variations in applicability, sensitivity, and resistance to environmental interference among different techniques, it is necessary to select detection methods based on site-specific conditions or integrate multiple technologies to improve the overall accuracy and reliability of the monitoring system.

## 3. Biological Characteristic Detection

In embankment termite detection, biological characteristic detection techniques primarily include gas detection and activity sign detection. Gas detection utilizes sensors to identify and measure specific gases, such as carbon dioxide and methane, released by termite metabolic activity, thereby indirectly indicating the presence and activity levels of termites [34]. Activity sign detection involves observing visible traces left by termites on the embankment structure’s surface or interior, such as mud tubes, nests, and erosion marks [77]. The biological characteristic detection methods provide maintenance personnel with a non-invasive and efficient monitoring tool, effectively ensuring the structural integrity and safety of the embankment.

### 3.1. Gas Detection

In the field of embankment termite detection, the most commonly used gas detection technologies include electronic noses and scent-detection dogs. The electronic nose is a device that uses an array of chemical sensors to analyze and identify trace gas components in the air. It is capable of detecting gases released by termite activity, such as methane and carbon dioxide, providing rapid and accurate detection results [35]. On the other hand, scent-detection dogs, with their highly sensitive olfactory system, can detect even trace concentrations of termite metabolic products. Their flexibility and convenience make them an important tool for on-site detection [78]. Therefore, the electronic nose is suitable for long-term monitoring and data analysis, while scent-detection dogs offer immediate response capabilities, making them ideal for emergency detection.

#### 3.1.1. Electronic Nose

Termite colonies emit a variety of gases, including CO_2_, CH_4_, CHCl_3_, N_2_O, CO, and H_2_, among which carbon dioxide (CO_2_) and methane (CH_4_) are the most common and abundant. The emission of these gases provides a theoretical basis for the non-destructive detection of termites [34,36]. Since the concentration of these gases increases with termite population size and activity levels, technicians can use gas-specific sensors to estimate the scale of a termite nest based on detected gas concentrations [79,80]. Electronic nose systems, which emerged in the 1980s, offer a promising non-invasive approach for detecting volatile organic compounds emitted by termites. These systems typically consist of a multi-sensor array, signal preprocessing units, pattern recognition algorithms, and a reference database. Upon interaction with VOCs, the sensors produce electrical signal changes, which are processed and analyzed to identify specific odor patterns associated with termite activity [81,82,83].

In current detection studies of termite pheromones and VOCs, three primary types of gas sensors are commonly employed, including metal oxide semiconductor sensors, CP sensors, and QCM sensors. Metal oxide semiconductor sensors offer fast response times and robust structural simplicity, making them well-suited for large-scale field deployment, particularly for real-time monitoring of broad-spectrum VOCs released by termites in outdoor environments such as levees and forested areas [37]. CP sensors provide better chemical selectivity and moderate sensitivity, allowing for the construction of distinct olfactory fingerprints, and are widely used in species classification tasks, as demonstrated in systems like the Aromascan A32S electronic nose [35]. QCM sensors, known for their exceptionally high mass resolution and sensitivity, are capable of detecting trace concentrations of specific pheromone compounds and are more suitable for high-precision detection in laboratory conditions or enclosed sensor platforms. These three types of sensors differ significantly in operating principles, environmental adaptability, deployment strategies, and detection performance. Therefore, properly integrating these three types of sensors into a multi-channel electronic nose array, the more efficient identification and early warning of termite activity can be achieved.

**Figure 6:**
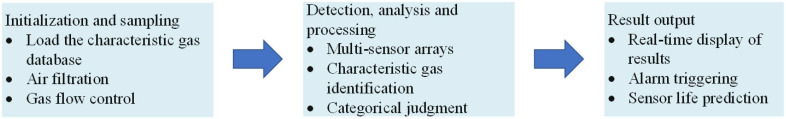
Workflow of termite detection using an electronic nose.

The operating process is illustrated in Figure 6, encompassing the entire procedure from sampling to data analysis and report generation. In an experimental study, Wilson et al. [35] employed the Aromascan A32S e-nose—comprising 21 chemical sensors—to investigate the odor signatures of four subterranean termite species. Their findings demonstrated that the system could effectively discriminate between species-specific VOC profiles, supporting its potential for species-level identification in termite monitoring. The application of e-nose systems in embankment environments offers several advantages. Most notably, they enable real-time, non-destructive detection of termite-related gas emissions, thereby reducing physical disruption to embankment structures. When integrated with artificial intelligence-based pattern recognition algorithms, such systems can achieve higher classification accuracy and robustness under variable field conditions.

However, the complexity of odor mixtures in open environmental settings, such as embankments, remains a significant challenge. Fluctuating ambient VOC levels caused by soil composition, microbial activity, and meteorological conditions can confound detection accuracy. Future research should also explore advances in sensing materials and device performance, in combination with sensor fusion and adaptive learning models, to enhance the reliability and scalability of electronic nose systems [84].

#### 3.1.2. Sniffer Dogs

**Figure 7:**
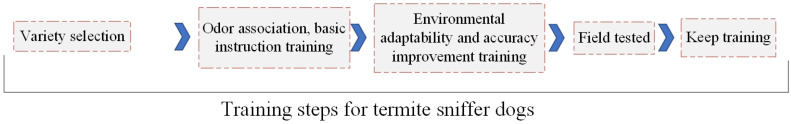
Workflow of termite detection using sniffer dogs.

Sniffer dogs have been widely applied in the detection of various insect species, including termites, owing to their highly sensitive olfactory capabilities [85,86]. Multiple studies have demonstrated that trained dogs, across different breeds, can achieve high detection efficiency in entomological surveys [87,88,89,90,91]. As illustrated in Figure 7, the training process typically involves systematic stages, including breed selection, specialized training, and performance evaluation, each requiring substantial time and resources.

In both complex building environments and extensive embankment structures, sniffer dogs have shown superior performance in detecting termite infestations that are difficult to identify through conventional human inspection methods [10,92]. Experimental studies have reported that the detection accuracy of trained sniffer dogs can be up to four times higher than that of experienced human inspectors [90]. Consequently, sniffer dogs have been incorporated into termite detection programs in various regions, demonstrating high operational effectiveness [93]. For example, Brooks et al. [78] achieved a detection accuracy of 95.93% using German Shepherds and Beagles to identify eastern subterranean termites.

However, despite their high detection accuracy, the practical application of sniffer dogs faces limitations. The high costs associated with training, certification, and maintenance restrict their widespread use, particularly in large-scale operations or regions with limited resources [94]. Moreover, environmental factors such as temperature, humidity, and background odors may influence the detection performance.

### 3.2. Termite Activity Sign Detection

The termite activity sign detection technologies primarily include visual inspection, intelligent monitoring, and drone-based termite detection. Visual inspection involves trained personnel conducting detailed on-site examinations to identify signs left by termites, such as mud tubes, boreholes, and structural damage [95]. While this method is straightforward and cost-effective, its accuracy largely depends on the inspector’s experience and observational skills. Intelligent monitoring leverages sensors and internet of things technologies to enable automated real-time data collection and analysis of termite activity [96]. This approach enhances detection efficiency, enables early warnings, and reduces reliance on manual inspections. Drone image analysis combines aerial photography with computer vision algorithms to scan large areas of the embankment surface for potential termite activity indicators [41]. This technique offers wide coverage, rapid data acquisition, and remote accessibility, making it suitable for difficult-to-reach or expansive embankment areas. The integration of these approaches offers a more comprehensive and proactive solution for detecting termite infestations in embankments. This not only strengthens early warning capabilities but also significantly contributes to maintaining the structural integrity and safety of embankment facilities.

#### 3.2.1. Visual Inspection

**Figure 8:**
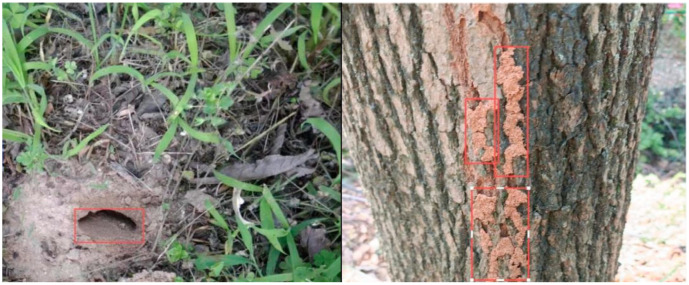
Typical signs of termite activity: mud tubes and small holes [43].

In embankment termite detection using visual inspection, attention should be focused on surface traces left by termite activity, such as termite mounds, swarming holes, and mud tubes, as shown in Figure 8. This method requires inspectors to possess substantial experience and professional judgment to accurately identify various forms of termite activity. During nest construction, termites reassemble soil particles using saliva and excreta, forming structurally stable mounds [77]. This process often increases the concealment of the surrounding environment, making detection more difficult. Therefore, in practice, thorough inspections of the embankment surface and key areas such as the platform and toe of the embankment are necessary to assess the distribution density of termite mounds and evaluate infestation severity [26,97].

Although visual inspection, as one of the earliest and most widely used detection methods, is low-cost and easy to implement, it has limitations. It may fail to detect nests located deep underground or within embankment structures and is highly dependent on the inspector’s experience and observational skills. Studies have shown that termites, during mound construction and maintenance, can regulate microbial abundance and diversity in specific regions of the mound [95,98,99,100]. As a result, environmental characteristics surrounding the mound—such as the presence of dense weeds, dead branches, and dry grass—are important indicators of termite activity and should be prioritized in monitoring efforts.

Termites, as typical environment-sensitive insects, exhibit survival and activity patterns that are largely regulated by external climatic conditions. Temperature and humidity not only directly affect their metabolic rates and feeding behaviors but also determine the spatial extent of nest construction, tunneling activities, and colony expansion [101]. Therefore, environmental factors, particularly fluctuations in temperature and humidity, are critical drivers influencing the intensity and distribution of termite activities. Xu et al. [102] demonstrated that termite excavation efficiency and maximum tunneling distance were significantly enhanced under conditions of moisture content below 10–15% and ambient temperature around 28 °C. Nakayama et al. [103] experimentally found that the feeding activities of two subterranean termite species were optimal at approximately 30 °C, and concluded that fluctuations in temperature and humidity had a significant impact on their behavior. Cornelius et al. [104] and Wiltz et al. [101] reported that subterranean termite activity increases during the spring and summer months, while significantly declining in winter as a result of lower temperatures. When conducting visual inspections, it is crucial to take these environmental factors into account in order to improve detection accuracy. This implies that inspection methods and focal areas should be adjusted seasonally or according to environmental conditions to more accurately assess and respond to termite infestations.

#### 3.2.2. Intelligent Monitoring

Intelligent monitoring systems represent a significant technological advancement. These systems primarily rely on advanced sensor technologies, wireless communication methods, and data processing algorithms, providing new tools for real-time monitoring and early prevention of termite infestations [31]. In practical applications, sensors are responsible for collecting various types of data related to termite activity, which is then reliably transmitted to cloud servers via wireless communication technologies. On the cloud server, specific data processing algorithms conduct in-depth analysis of the collected data, thereby delivering accurate early warning information about termite activity [59,67].

**Figure 9:**
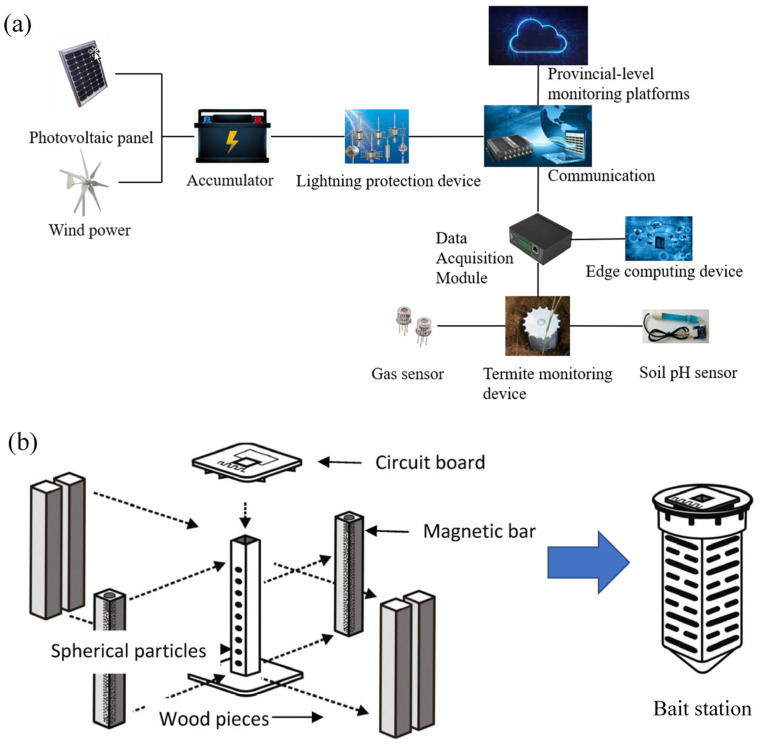
Intelligent monitoring system. (**a**) Schematic diagram of the intelligent monitoring system. (**b**) Electromagnetic induction bait station [49].

In recent years, the application of non-loop electromagnetic induction methods in termite monitoring has made significant progress. One study demonstrated that with the help of a handheld reader and a specific termite bait station shown in Figure 9b, the detection rate reached 97.5–98.5%, providing an important approach for remote termite surveillance [49]. Meanwhile, research by Ming Wang et al. [42] introduced an intelligent monitoring technology based on termite feeding behavior and environmental CO_2_ concentration, with its working principle illustrated in Figure 9a. This technology exhibits excellent waterproof and anti-interference performance, achieving a monitoring accuracy of over 90%. Although these technologies show great potential, future research still needs to explore the applicability and cost-effectiveness of various methods to better adapt to different environments and develop solutions with higher precision and reliability.

#### 3.2.3. Drone-Based Termite Detection

Traditional termite detection methods mainly rely on manual inspection and expert experience. However, termite nests are often well-concealed, making this approach not only inefficient but also prone to inaccuracies in detection results. In recent years, drones have emerged as highly efficient tools for data collection. Drone-based termite detection technologies in embankments have become a promising and innovative approach with significant potential [105]. Multi-temporal image comparison is a promising approach to enhance the effectiveness of UAV-based termite detection methods. During the initial deployment phase, UAV flights can be scheduled on a monthly or bi-monthly basis to capture early surface anomalies such as termite mound formation or vegetation stress. In the routine monitoring stage, a quarterly inspection frequency is recommended to track the seasonal dynamics of termite activity. Previous studies have shown that subterranean termite activity increases significantly during spring and summer, while declining noticeably in winter due to lower temperatures [101,104]. Therefore, flight intervals should be adjusted according to seasonal and environmental changes to achieve a more efficient and cost-effective termite detection strategy.

**Figure 10:**
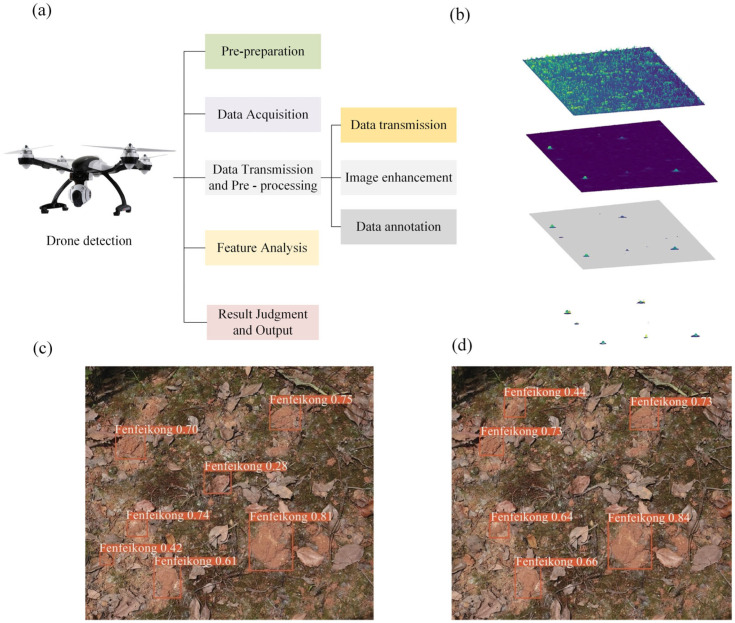
Principle and application of drone-based termite detection. (**a**) Detection workflow. (**b**) Image feature extraction [41]. (**c**) Before integration of adaptive color perception module [96]. (**d**) After integration of adaptive color perception module [96].

Drones offer a key advantage in their flexibility to be equipped with a variety of devices as needed. As illustrated in Figure 10a, they can carry high-definition cameras, multispectral sensors, and thermal infrared cameras [106]. By analyzing the high-resolution images of embankment surfaces collected by these devices, drones can identify typical signs of termite activity, such as mud tubes, mud lines, and swarming holes. D’hont et al. [41] utilized a RIEGL RICOPTER drone equipped with a VUX-1UAV scanner (RIEGL Laser Measurement Systems GmbH, Horn, Austria) to detect termite mounds in Australia, as shown in Figure 10b. Their study achieved a detection accuracy of 81%, demonstrating that drone-based light detection and ranging scanning can accurately locate termite mounds and extract detailed 3D information such as height and volume. The data acquired can be used to accurately estimate the size and structure of termite colonies, offering a foundation for further research and control strategies.

In the application of image recognition algorithms, both machine learning and deep learning algorithms have been widely adopted to make the image analysis process more efficient and accurate [43]. Sandino et al. [107] developed an automated termite mound detection method by integrating UAV-based hyperspectral imaging, support vector machine classification, and shape-based feature analysis, achieving efficient identification and localization of termite mounds with an overall accuracy of 68%. Wang et al. [96] developed an improved one-stage object detection algorithm called ACP-YOLOv5s. This model integrates an adaptive color perception module to optimize performance in complex natural environments by effectively enhancing feature extraction and color perception capabilities. The comparison results are shown in Figure 10c,d, demonstrating a significant improvement in accuracy. Zhang et al. [108] developed an improved UAV small object detection approach based on the YOLOv8 algorithm, which resulted in notable improvements in detection performance, with increases of 17.2%, 10.5%, and 16.2% in mean average precision, precision, and recall, respectively.

Research in image recognition has made significant contributions to the identification of termite activity, enhancing the intelligence level of termite monitoring by improving accuracy while reducing manpower. However, the complex environmental background surrounding embankments—such as vegetation cover and soil texture—can interfere with image recognition algorithms, potentially leading to false positives or missed detections. Therefore, it is necessary to further optimize UAV hardware performance and image recognition algorithms to enhance detection accuracy and stability under complex environmental conditions. Strengthening multi-technology integration, such as combining UAV imagery with ground sensor data, can help achieve comprehensive and precise detection of termite activity in embankment structures.

### 3.3. Summary

**Figure 11:**
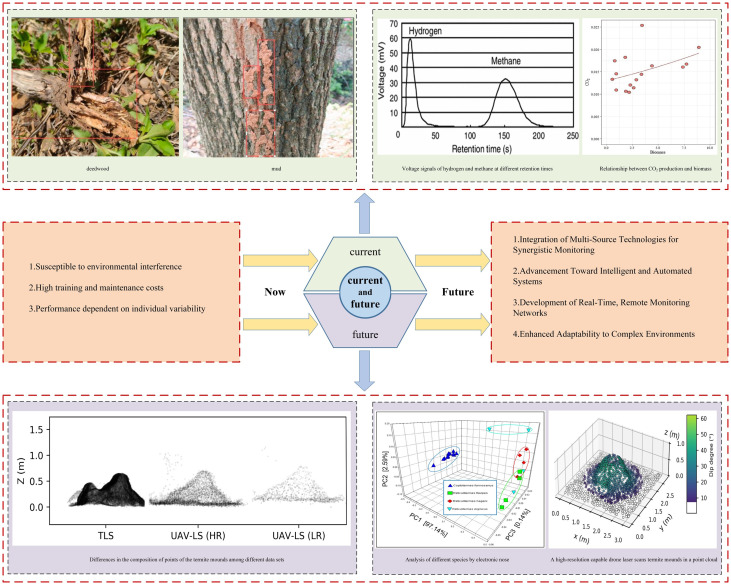
Development trends of biological characteristic-based detection technologies [35,41,43,80,109].

In embankment termite detection, biological characteristic detection technologies offer an efficient and non-invasive monitoring means by identifying biological markers such as odors and behavioral traits associated with termite activity. Common technologies include electronic noses, sniffer dogs, and drone-based detection methods. As shown in Table 3, several biological characteristic detection methods were systematically evaluated across key dimensions such as resolution, environmental limitations, cost, deployment complexity, and data interpretation. The electronic nose uses high-precision sensors to detect volatile compounds released by termites, making it suitable for large-scale monitoring, especially in environments with minimal interference. However, its accuracy can be affected by environmental odors. Sniffer dogs, with their exceptional olfactory abilities, can accurately detect termite activity hidden in complex terrain or concealed areas, making them ideal for high-precision local detection, though the high cost of training and operation limits their widespread use. UAV-based image analysis, equipped with high-resolution cameras and combined with artificial intelligence, allows for large-scale monitoring of embankment surfaces, effectively identifying visible signs of termite activity, such as termite mounds and mud tubes, especially in large or hard-to-reach areas. Despite the high flexibility and adaptability of biological detection technologies, challenges such as environmental conditions, equipment performance, and operational settings still exist in practice. Figure 11 summarizes the current research deficiencies and future research directions in biological characteristic detection. Therefore, visual inspection and intelligent monitoring are often integrated with biological detection technologies to fully leverage their advantages. Visual inspection is suitable for routine patrols and preliminary assessments, while intelligent monitoring systems enable real-time, automated termite surveillance around the clock. By combining these techniques, detection accuracy and efficiency can be greatly improved, providing comprehensive technical support for the prevention and management of termite infestations in embankments.

## 4. Discussion

Environmental variables such as soil moisture, surface roughness, and temperature fluctuations critically influence the reliability of termite detection in embankments. In particular, GPR performance deteriorates in high-conductivity or saturated soils, where signal attenuation and scattering hinder subsurface anomaly resolution [110]. Cui et al. demonstrated that wet clay-rich environments significantly limit the detection depth and resolution of GPR, though planar beam-array optimization can provide marginal improvement [38]. Remote estimation of soil dielectric properties, as explored by Álvarez López et al., offers a means to pre-characterize GPR suitability before field deployment [111].

Acoustic sensing is equally susceptible to ambient interference. Studies by Tejera-Berengue et al. revealed that wind speed, terrain clutter, and UAV distance introduce considerable noise into acoustic data, reducing classification accuracy. These challenges are especially relevant when attempting to detect termite feeding or movement signatures near vegetated embankment surfaces [112,113]. Controlled deployment windows and directional microphones have been proposed to mitigate such limitations [114]. Chemical sensors, such as electronic noses for detecting termite-emitted VOCs, are heavily affected by microclimatic shifts. Elevated temperature and humidity accelerate gas dispersion, compromising detection specificity. A multi-scenario model proposed by Qu et al. applies adaptive calibration to improve sensor stability across variable field conditions [115].

In more extreme field settings, such as saline or arid embankments, electrical resistivity methods outperform electromagnetic techniques by maintaining contrast under fluctuating ionic content. Field studies by Xiao et al. and Sørensen et al. confirm the stability of resistivity inversion techniques when monitoring subsurface features in high-salinity soils [116,117]. Additionally, terrain roughness—common along natural or aging levees—can disrupt both sensor placement and UAV imaging stability. As shown by Sørensen et al., automated passive sensing systems often struggle with localization and noise control in uneven landscapes [118].

**Figure 12:**
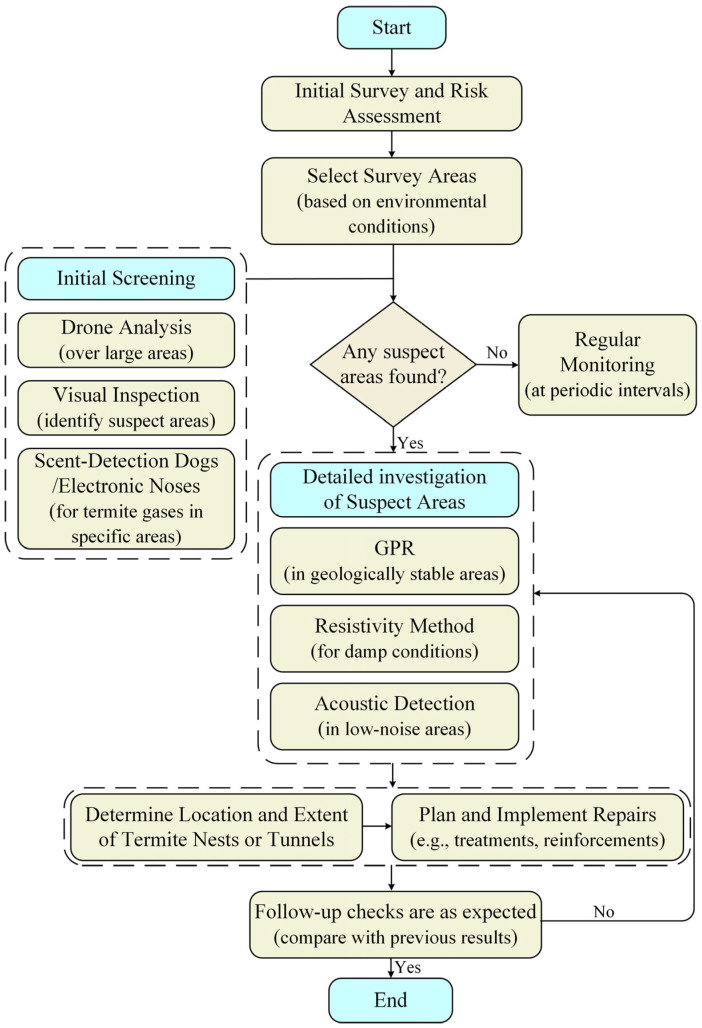
Flowchart of multi-sensor detection methods.

In summary, termite detection systems must be tailored to site-specific environmental conditions. By integrating multi-sensor detection technologies, such as combining UAV imagery with GPR or acoustic sensing, the limitations of each modality can be offset, resulting in more accurate and robust monitoring results for embankment infrastructure. Meanwhile, improvements in both the materials and overall performance of key equipment can more effectively mitigate uncertainties caused by environmental factors, thereby enhancing the accuracy and reliability of detection in future applications [84,119,120]. Figure 12 gives an implementation roadmap that integrates multi-sensor detection methods, covering the process from survey assessment to follow-up inspection.

## 5. Conclusions and Outlook

In recent decades, embankment termite detection technologies have made significant progress, with a variety of physical and biological feature-based methods widely applied in engineering practices. As shown in Table 4, physical detection techniques such as GPR, acoustic testing, and resistivity methods allow for accurate localization of termite nests and tunnels without damaging the embankment structure, offering advantages in precision and real-time response. Meanwhile, biological detection technologies—such as electronic noses, sniffer dogs, and UAV-based image analysis provide efficient and non-destructive means of identifying termite activity by detecting emitted odors or surface activity signs. However, due to environmental influences, the effectiveness of these detection methods may vary under different conditions, as reflected in Single detection techniques often have limitations. For instance, GPR is greatly affected by geological conditions, acoustic testing can be disrupted by ambient noise, and electronic noses and sniffer dogs may be influenced by environmental odors. Therefore, practical applications typically require the integration of multiple detection technologies. For example, preliminary identification of termite hotspots can be conducted using visual inspection, UAV imagery, electronic noses, and sniffer dogs. Subsequently, large-area scanning is performed using GPR, followed by acoustic or resistivity methods to confirm the termite nest location and scale accurately. Additionally, it is important to adjust detection strategies based on seasonal variations and structural characteristics of different embankment sections.

GPR can be used to identify subsurface anomalies such as voids and loosened soil caused by termite nesting activities. Meanwhile, acoustic sensors deployed on or within embankments are capable of capturing subtle vibrations generated by termite feeding or movement. The spatial and functional complementarity of these two methods enables effective cross-validation of anomalous signals, thereby significantly reducing the false alarm rate. In addition, UAVs equipped with high-resolution cameras allow for rapid large-area inspection of embankment surfaces, detecting external signs of termite activity such as vegetation wilting due to root damage, mud tubes, mud lines, and swarming holes. However, these surface indicators may be obscured by vegetation or remain imperceptible in the early stages. To overcome this limitation, we recommend deploying baited, miniaturized VOC sensors at fixed key locations. These sensors can detect termite-released pheromones or microbial metabolites (e.g., methane) emitted from active nests. By integrating UAV image analysis with VOC detection data, high-risk areas can be more accurately identified, enabling ground personnel to conduct targeted inspection and intervention.

Over the next few years, enhancing the accuracy and robustness of individual detection technologies remains a critical focus. Efforts should be directed toward improving the anti-interference performance of acoustic detection systems, particularly in complex and noise-prone environments such as embankments adjacent to traffic or water flow. Additionally, the development of multi-source data fusion frameworks—combining ground-penetrating radar, acoustic sensing, and electronic nose technologies—can significantly improve the comprehensiveness and reliability of termite detection. To support such integration, artificial intelligence algorithms, particularly those for pattern recognition and anomaly detection, should be optimized for the processing of heterogeneous sensor data. These improvements will enable the early identification of infestation zones with greater confidence and minimal disruption to embankment structures. Moreover, practical engineering applications would benefit from the development of standardized operational protocols that streamline detection sequences and facilitate technology deployment in the field.

In the long term, the future of termite detection in embankments lies in the realization of fully automated, intelligent monitoring systems powered by advanced sensing technologies and AI-driven analytics. A key research direction involves deploying UAVs equipped with hyperspectral imaging and thermal infrared sensors, coupled with deep learning-based object detection algorithms, to enable large-scale, high-resolution surveillance of embankment surfaces. These systems can autonomously identify early-stage signs of termite activity—such as mud tubes or thermal anomalies—and generate real-time detection reports, reducing the reliance on manual inspection.

Furthermore, the integration of sensor networks into Internet of Things architectures will facilitate the construction of real-time, continuous monitoring platforms. Such platforms are expected to enable 24/7 surveillance and predictive maintenance capabilities, ultimately shifting termite management strategies from reactive to proactive. In the long term, research should also explore cross-disciplinary approaches that combine geophysics, entomology, environmental science, and data engineering to foster innovation in both hardware design and algorithmic modeling. By embracing this holistic framework, the field can advance toward the goal of highly intelligent, adaptive, and resilient embankment monitoring systems, ensuring the long-term structural safety and ecological sustainability of critical infrastructure.

## Figures and Tables

**Table 1:** Extensive applications of GPR in embankments.

Country	Location	GPR Model	Measurement Length (m)	Frequency	Detection Depth (m)	Data Processing	Conclusions	References
USA	Lollie Levee	GSSI SIR-30 GPR (Geophysical Survey Systems, Inc. (GSSI), Nashua, NH, USA)	850	900 MHz, 400 MHz	0.5–1, 2–3	GSSI RADAN7	The resolution is related to the detection depth.	[40]
USA	London Avenue Canal	SIR-3000 GPR (Geophysical Survey Systems, Inc. (GSSI), Nashua, NH, USA)	4442	400 MHz	0.61	SAS 9.1	Detect tree roots, internal voids, and termite nests in embankments.	[56]
China	Nanmenxia Reservoir	Groundvue (Utsi Electronics Ltd., Cambridge, UK)	50	15, 50,400 MHz	70,22,5	ReflexW	Seepage analysis.	[32]
Italy	Medau Zirimilis	-------	480	100, 250, 500 MHz	10,4,2	CUI-2 Central Unit	Detection of cracks and seepage zones.	[50]
Italy	Reno River	RIS MF (IDS) (IDS GeoRadar s.r.l., Pisa, Italy)	500	100, 200, 500, 600 MHz	3.5–4, 2–3	-------	Detection of cavities.	[57]
Italy	Travallino, Lousana	RAMAC/GPR (Mala Geosciences Co., Stockholm, Sweden)	-------	250 MHz	0.4–30	ReflexW 5.0	Locating cavities.	[58]

**Table 2:** Comparison of GPR, acoustic methods, and ERT in termite detection.

Parameter	Ground-Penetrating Radar	Acoustic Detection	Electrical Resistivity Tomography
Physical Principle	Electromagnetic wave reflection [2].	Detection of transient mechanical (vibration) signals [66].	Subsurface electrical resistivity measurement [69].
Resolution	Moderate to high (depends on frequency) [2].	High (for localized impulsive events).	Low to moderate (meter-scale resolution).
Environmental Limitations	Affected by salinity, clay, and high moisture [2].	Sensitive to wind, vibration, background interference [31,59].	Requires good ground contact, susceptible to temperature extremes.
Deployment Complexity	Moderate (portable, surface scanning).	Low (requires surface-mounted sensors) [31,59].	High (electrode array setup, contact-dependent) [44].
Automation Readiness	Moderate (integratable with robotic platforms).	Moderate (requires signal processing) [31]	Low (mostly manual setup and control).
Cost	Moderate to high.	Low to moderate.	High (equipment and field deployment).
Data Interpretation	Moderate (requires signal inversion and depth calibration) [48].	Moderate to complex (needs filtering and feature extraction) [31,59].	Complex (inversion modeling, geological expertise needed) [44].
Typical Application	Detecting cavities, tunnels, or moisture anomalies near surface [2].	Real-time detection of termite activity in confined media.	Mapping of large-scale termite-affected zones and subsurface heterogeneity [44].

**Table 3:** Comparison of electronic nose, intelligent monitoring, drone image analysis and sniffer dogs in termite detection.

Parameter	Electronic Nose.	Intelligent Monitoring.	Drone Image Analysis.	Sniffer Dogs.
Physical Principle	VOC pattern recognition via gas sensor arrays [35].	Data fusion from multi-sensor networks and machine learning.	Remote imaging [41].	Olfactory detection of termite-related scents [78].
Environmental Limitations	Sensitive to temperature and humidity changes.	Robust when properly configured and shielded.	Wind, rain, fog, and canopy cover limit performance.	extreme temperatures affect dog performance; heavy rain and snow limits scent detection; dense vegetation may block scent paths.
Deployment Complexity	Low (portable, battery-powered, field-friendly).	High (requires sensor integration, network, AI backend) [42,49].	Moderate (requires flight clearance, GPS setup).	Low–moderate (needs handler training, dog conditioning; limited by dog endurance/availability) [78].
Automation Readiness	High (pattern recognition and real-time output).	Very high (autonomous decision-making and cloud control) [49,92].	High (predefined flight and onboard image processing possible) [41].	Low (relies on human handler interpretation of dog behavior) [78].
Cost	Moderate (low hardware cost, requires calibration).	High (sensors and infrastructure and software).	Moderate–high (drone, sensors, training costs) [106].	Low–moderate (dog acquisition/training, ongoing care) [43].
Data Interpretation	Simple with AI model; requires calibration dataset [35].	Complex; AI models must be trained per site [42].	Moderate; machine learning model required for image classification [41,96,108].	Subjective (handler interprets dog alerts; experience-based) [78].
Typical Application	On-site early-stage termite gas detection (VOCs) [35].	Long-term continuous monitoring of termite activity and risk [42].	Large-area inspection of termite mounds or infestation signs [41].	Targeted on-ground search for active termite colonies, nest localization in complex terrains [78].

**Table 4:** Comparison of different detection methods.

Detection Technology	Advantages	Disadvantages	Application Scenarios	Detection Accuracy
Ground-penetrating radar	Non-invasive, real-time data, high precision.	High cost, susceptible to geological conditions.	Large-area preliminary detection.	96% [29]
Acoustic detection	Non-destructive, fast detection.	Highly affected by noise environment.	Relatively quiet local detection.	98.316% [65]
Electrical resistivity method	Capable of estimating nest location and volume.	Limited detection depth.	Areas with relatively stable geological conditions.	-
Electronic nose	High precision, real-time analysis and processing, remote monitoring.	Easily affected by ambient odors, high cost.	Large areas with minimal interference.	72.7% [78]
Sniffer dogs	Strong adaptability to complex environments, able to detect trace odors.	High training costs, influenced by the dogs’ physical condition.	Complex terrains, high accuracy requirement areas.	95.93% [36]
Visual inspection	Low cost, more intuitive.	High subjectivity, influenced by season.	Routine patrols, preliminary judgment.	-
Intelligent monitoring	High real-time performance, labor-saving, predictive.	High cost, easily affected by the environment.	Areas with minimal interference.	97.5–98.5% [42]
Drone image analysis	Wide coverage, high efficiency, monitoring of hazardous areas, capable of dynamic comparison across periods.	Poor performance in early detection, weather-dependent, relies on image processing algorithms and technology.	Large-scale macro monitoring.	81% [97]

## Data Availability

Not applicable.

## Abbreviations

The following abbreviations are used in this manuscript:

CP	Conductive polymer
ERT	Electrical resistivity tomography
GPR	Ground-penetrating radar
QCM	Quartz crystal microbalance
UAV	Unmanned aerial vehicle
VOC	Volatile organic compound
